# Effectiveness of sexual health influencers identified by an ensemble machine learning model in promoting secondary distribution of HIV self-testing among men who have sex with men in China: study protocol for a quasi-experimental trial

**DOI:** 10.1186/s12889-021-11817-2

**Published:** 2021-09-28

**Authors:** Ying Lu, Yuxin Ni, Qianyun Wang, Fengshi Jing, Yi Zhou, Xi He, Shanzi Huang, Wencan Dai, Dan Wu, Joseph D. Tucker, Hongbo Jiang, Liqun Huang, Weiming Tang

**Affiliations:** 1 Dermatology Hospital of South Medical University, Guangzhou, China; 2 University of North Carolina Project-China, Guangzhou, China; 3 Institute for Healthcare Artificial Intelligence, Guangdong Second Provincial General Hospital, Guangzhou, China; 4 School of Data Science, City University of Hong Kong, Hong Kong, SAR China; 5 Zhuhai Center for Diseases Control and Prevention, Zhuhai, China; 6 Faculty of Medicine, Macau University of Science and Technology, SAR, Macau, China; 7 Zhuhai Xutong Voluntary Services Center, Zhuhai, China; 8 London School of Hygiene and Tropical Medicine, London, UK; 9 Guangdong Pharmaceutical University, Guangzhou, China

**Keywords:** Sexual health influencers, Ensemble machine learning, Secondary distribution, HIV self-testing, Men who have sex with men, China

## Abstract

**Background:**

HIV self-testing (HIVST), especially the secondary distribution of HIVST (SD-HIVST) initiated by sexual health influencers (SHIs), has been recognized as an effective strategy in promoting HIV testing, especially among men who have sex with men (MSM). This quasi-experimental study aimed to evaluate whether SHIs identified through the ensemble machine learning approach can distribute more HIVST than those who identified by the empiricalscale.

**Methods:**

We will recruit eligible adults (≥18 years old) who were assigned male gender at birth, and willing to participate in potential SD-HIVST online. Participants will be assigned randomly to two groups (scale group or machine learning group), followed by a separate process of SHI identification based on the group assignment. After identification, all index participants (defined as identified SHIs who are verbally consented to participate in SD-HIVST or who directly order HIVST kits) will follow the same procedure for SD-HIVST acquisition and distribution. Index participants can order HIVST online and distribute them to members within their social networks (defined as alters) in-person or virtually through a personalized peer referral link. Once a unique alter uploads a photographed test result to the platform, both the alter and the corresponding index participant will receive a fixed incentive of 3 USD. The index MSM can order up to five HIVST in the first three months and ten HIVST in the following three months. Each index participant will need to complete a baseline survey at the first-time ordering and one to two follow-upbased on the times of ordering,, three months after ordering. This trial will be comparing 1) the mean number of alters motivated by each index participant in each group and 2) the mean number of newly-tested alters motivated by each index participant in each group.

**Discussion:**

In promoting the efficacy of identifying SHIs for SD-HIVST, our study has the potential to enhance testing coverage, particularly among marginalized individuals and those who are reluctant to for HIV and other sexually transmitted infections.

**Trial registration:**

We registered the study on the Chinese Clinical Trial Registry website on 4th November 2021, with registration number ChiCTR2000039632.

## Background

The global human immunodeficiency virus (HIV) epidemic has been well controlled [1, 2]. However, study findings still reinforce the need for further attention to men who have sex with men (MSM) regarding HIV prevention. For instance, the HIV prevalence among MSM in China has increased from 0.5% in 2003 to 8.0% in 2015 and stays consistent afterward, while this also consistently stands highly of HIV incidence in China [3–5]. The lack of HIV status awareness is one of the main identified reasons for the high HIV infection rate among the MSM population [1]. Unfortunately, a recent statistic showed that more MSM in China (about 40–50%) than in other developed countries are less likely to have tested for HIV in the last 12 months [6]. Therefore, HIV testing is considered the key and the first step needed in HIV transmission prevention, specifically among MSM [7]. Following testing, the need for successful linkage to care and treatment for all diagnosed persons living with HIV is also quite essential.

Compared with healthcare facilities-based tests, HIV self-testing (HIVST) affords users greater convenience, autonomy, and privacy [8, 9]. HIVST is recognized globally as an innovative and reliable strategy to promote HIV testing. The World Health Organization (WHO) also recommends HIVST as an efficient testing approach for persons with low access to testing services and high-at-risk persons for routine testing [10]. Secondary distribution of HIVST kits (SD-HIVST) refers to a strategy where individuals (defined as index) obtain multiple HIVST kits and distribute them to members within their social networks (defined as alters) [11]. Currently, a growing number of empirical pieces of evidence have shown that SD-HIVST could contribute to the increasing coverage of HIV testing and effectively promote HIV case identification in diverse populations [12–14].

In addition, individuals within centralized networks could contribute to health behavior change among their peers [15] and increase the distribution of HIV self-testing kits [16] through SD-HIVST. How to effectively identify sexual health influencers (SHIs) among MSM is relevant in implementing SD-HIVST interventions. However, most preliminary studies employ self-reporting or empirical scales that cannot evaluate SHIs’ social network characteristics and have limited influence [17–21]. Therefore, we developed an ensemble machine learning model to help identify key SHIs [22]. This quasi-experimental trial sought to examine if SHIs identified through our trained ensemble machine learning model can facilitate more testing among persons who receive HIVST in the SD-HIVST intervention.

### Objectives

This trial aims to examine whether the SHIs identified by an ensemble machine learning model can motivate more alters to receive HIVST kits and more first-timers to test for HIV, compared to SHIs identified through an empirical scale.

### Hypothesis

We hypothesize that SHIs identified by the ensemble machine learning model will motivate more alters to test and identify more newly-tested alters (as first-time tested for HIV) through the SD-HIVST intervention than SHIs identified by the conventional scale.

### Trial design

This trial will be a double-blinded, quasi-experimental study with two parallel groups. The study duration is 12 months, from January 2021 to December 2021. We will randomly assign study participants into two groups in a 1:1 allocation ratio after recruitment. After that, we will use two distinct strategies (model-based and scale-based methods) in identifying the SHIs within each group who will receive the same intervention (SD-HIVST).

## Methods/design

### Study setting and recruitment

The study will be conducted jointly by the Social Entrepreneurship to Spur Health (SESH) team, Zhuhai Center for Diseases Control and Prevention (CDC), and Zhuhai Xutong Voluntary Services Center (Xutong), which is an MSM-friendly community-based organization (CBO).

We will post recruitment banner adverts on Blued, the largest online gay social networking application among MSM in China, in 5 provinces of southern China (Guangdong, Guangxi, Fujian, Hunan, Jiangxi). Interested MSM who click the banner posts will undergo an eligibility assessment using a set of pre-survey questions, and eligible participants will proceed to complete an online baseline survey.

### Eligibility criteria

To participate in the study, interested individuals are must meet the following criteria: 1) be assigned male gender at birth; 2) be at least 18 years or older; 3) self-report ever having sex with other men, and 4) be willing to participate in a potential secondary distribution of HIVST intervention.

### Interventions

We will randomly allocate eligible participants to either the scale group as controls or machine learning groupas the intervention group. A statistician will identify SHIs in the intervention group using an ensemble machine learning model and using a conventional scale for identification in the control group. All identified SHIs will be eligible to participate in the SD-HIVST intervention as index participants. We define index participants as those who were consented to participate in the program.

**Fig. 1:**
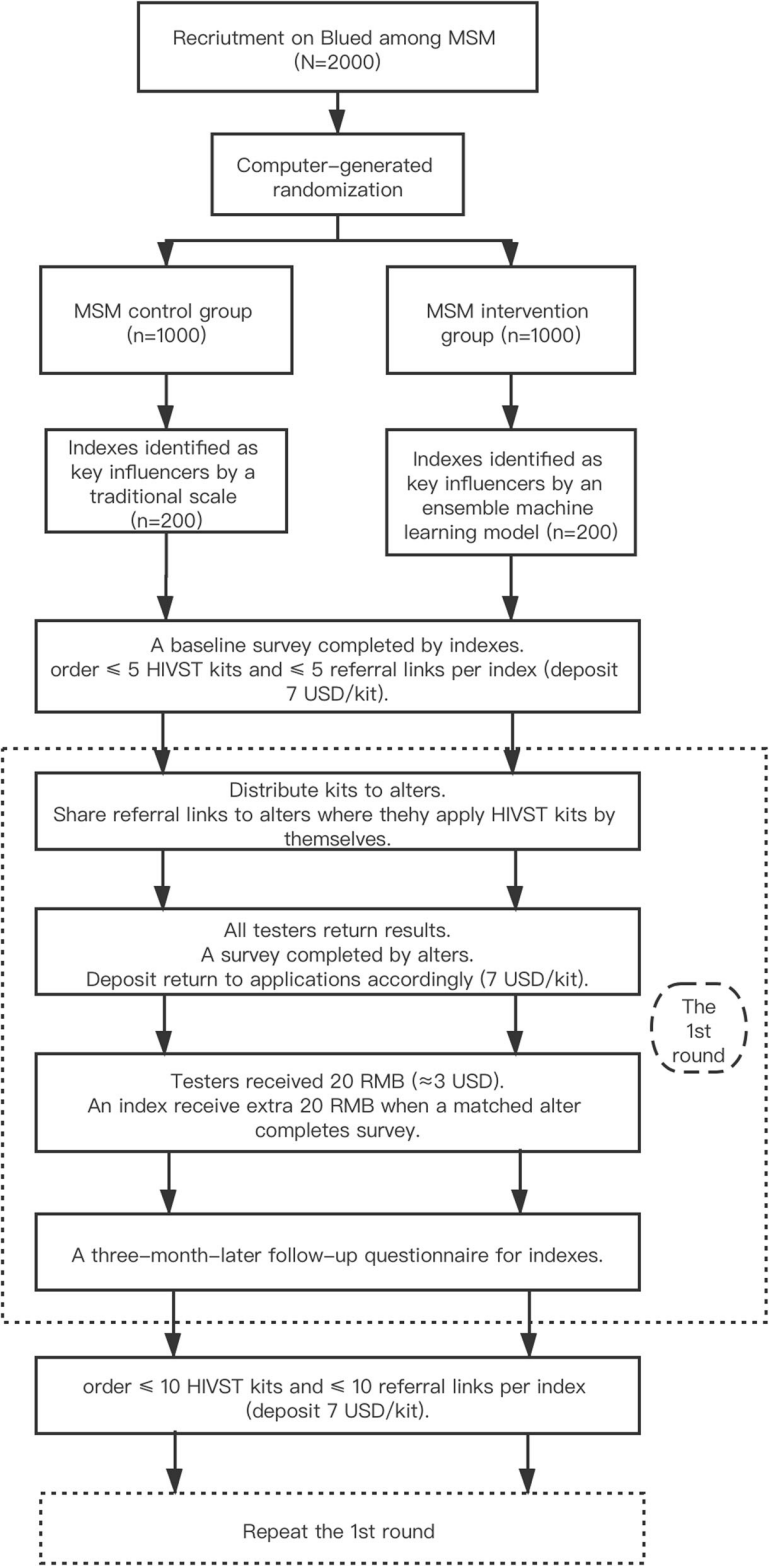
Trial Flowchart

Index participants from both groups will follow the same study procedures for SD-HIVST with monetary incentives plus peer referral. They will be required to complete a survey at the beginning of the first-time ordering and one follow-up survey three months after each time they order HIVST kits. Figure 1 is the flowchart which shows the trial procedures.

#### The scale group/ control group

SHIs among MSM participants assigned to the empiricalscale group (hereafter, scale group) will be determined using a 6-item scale. The same scale has been used by our team in a previous study [22, 23] to measure men’s sexual health influence, with a Cronbach alpha of 0.937. The scale consists of six questions that will assess each participants’ level of contribution in spreading HIV and sexually transmitted infections (STI) related information both online and offline in the past three months. The six questions include 1) how often they talked to others about HIV and STI; 2) how much information about HIV and STI they provided to others; 3) how many people they told about HIV and STI; 4) how likely they were to ask for more information about HIV and STI; 5) who communicated more information about HIV and STI: the participants or others within their social network; 6) how often they were to ask for advice about HIV and STI. Total response scores from answering the questions range from 0 to 24. Table 1 presents a detailed summary of responses to each question.

**Table 1:** Summarized responses of each item in the conventional scale

Typical item	Responses Categories	Method of scaling
In the past 3 months, how often did you talk to others about HIV and STI (including both online and offline)?	Never, seldom, sometimes, often, always.	5-point Likert scale, from 0 (never) to 4 (always).
In the past 3 months, how much information about HIV and STI did you provide to others (including both online and offline)?	No information provided, provided with a little information, provided with some information, provided with much information, provided with massive information.	5-point Likert scale, from 0 (no information provided) to 4 (provided with massive information).
In the past 3 months, how many people did you tell about HIV and STI (including both online and offline)?	None, few people, some people, many people, a lot of people.	5-point Likert scale, from 0 (none), 4 (a lot of people).
In the past 3 months, how likely were you to be asked for more information about HIV and STI (including both online and offline)?	Very unlikely, unlikely, neutral, likely, very likely.	5-point Likert scale, from 0 (very unlikely) to 4 (very likely).
In the past 3 months, who communicated more information about HIV and STI: you or others within your social network (including both online and offline)?	All information was told by others, most of information was told by others, information that others told me about was as much as I told them, most of information was told by me, all information was told by me.	5-point Likert scale, from 0 (all information was told by others) to 4 (all information was told by me).
In the past 3 months, how often were you to be asked for advice about HIV and STI (including both online and offline)?	Never, seldom, sometimes, often, always.	5-point Likert scale, from 0 (never) to 4 (always).

We may eliminate some top-point indexes (by either 20% or 30%, or other percentages) depending on the actual situation we encounter during the data collection. Also, the machine learning identification of the number of SHIs will be according to the ranking descending from the highest total points. We will randomly select the required number of indexes amongst the eligible SHIs with the lowest scale total scale point if more indexes share the same total scale point score. For example, if we would like to cut off the top 35 indexes out of 100 participants, however, 27 indexes scored at least 20 points, and the subsequent 12 indexes (Rank No. 28 to Rank No. 39) score 19 points, then we will randomly select eight indexes among the 12 indexes (i.e., No. 28 to No. 39). That will ensure that the cut-off percentage of 35% predefined before the selection will remain equal to the same number of machine learning identification results.

#### The machine learning group/ intervention group

SHIs in the intervention group will be identified using an ensemble machine learning model, which we have trained using our previous SD-HIVST intervention trial data [24]. The data from our prior SD-HIVST intervention pilot served as our training and validation data set. And the identification of the results (i.e., classification accuracy) of our proposed machine learning model outperformed the conventional scales cut-off method [22].

We determined significant predictors according to machine learning algorithms, using training dataset and validation dataset in our previous modeling study [22]. Thus, we will include questions regarding these predictors in the baseline survey. We will collect data on these selected predictors and input them into our pre-trained ensemble machine learning model. Output results from the model will show who the SHIs are in the machine learning group. If some predictor variables have unavailable or missing data, our model will still work as we can regard those data as ‘NA’ data in modeling input for prediction. Also, we trained three ensemble machine learning models in our previous modeling study for identifying SHIs. The models could distinctly predict key distributors (SHIs who are likely to distribute at least two kits to alters), key promotors (SHIs who can promote HIV testing among the first-time tested alters), and key detectors (SHIs who can contribute to positive alters detection). However, we found that the model of key distributors is also capable of identifying most of key promotors. Therefore, in this study, we will utilize the model of key distributors for SHI identification in the machine learning group as our main outcomes focus on the ability of index on motivating alters and newly-tested alters.

#### Procedure of secondary distribution of HIVST

All index participants will follow the one standard set of instructions and procedures for network-based online SD-HIVST regardless of group assignment. This procedure has been used in our previous studies and has shown effectiveness in increasing HIV testing coverage among Chinese MSM [24–26]. Ordering of HIVST kits and uploading test results will be all conducted using an online digital platform created and operated by a community health services center. Index participants can access this platform by following the public account of Xutong on WeChat, which is the largest social networking application in China.

Alters will be encouraged to perform all HIVST according to the manufacturers’ instructions using fingerstick blood samples. We will use SD Bioline HIV/Syphilis duo test kits (SD Bioline Company, South Korea). Each HIVST kit will have a delivery-tracking code, a result-tracking code, and a user guide manual attached to it. HIVST kits will be delivered to the destination address provided by applicants at request by post. The trained CBO staff verifies results based on photos uploaded by testers. Testers will be required to submit the testing evidence and test results by uploading photographs of their used kits to an online platform. That platform will be an anonymous system that testers can only access by scanning a QR code attached to their test kit box.

There will be two rounds for index participants to order HIVST kit(s) via the online platform. During the first three months, index participants can request at most five (5) HIVST kits based on their personal needs. They can personally use the kits or distribute them to others within their social network (hereafter, alters). In addition, index participants can order a personalized peer referral link, with which they can invite up to 5 unique alters to order one HIVST kit each. We will employ a refundable deposit strategy to reach a considerable response rate and facilitate linkage to care services. We will demand a refundable deposit amount of 40 RMB (≈ 7 USD) at each request. The deposit will be returned to the matched kit applicant through the same payment method within three business days after an uploaded testing result is verified by CBO staff.

All index participants will receive notification to order the second round of HIVST kits after completing the 3-month follow-up survey. Index participants who do not finish the 3-month follow-up survey will receive a survey reminder alongside the notice of the second round. For index participants who do not order any test kits during the first round, we will notify them about the second round when the first index participant completes the first follow-up survey. The only difference between the two rounds is that an index participant can be allowed to order up to 10 HIVST kits (5 HIVST kits in the first round) and can invite up to 10 unique alters (5 alters in the first round) to order one kit via the peer-referral link during the second round.

For results verification, trained CBO staff will first read test results according to uploaded photos. If a result is determined to be invalid, we will contact the tester via WeChat or phone call and ask whether the tester is willing to get re-tested. If a result is HIV-reactive or syphilis-reactive, the CBO staff will contact the teste to provide linkage to care and support services. The CBO staff will also verify the alters’ identification (by checking if the telephone number and WeChat ID provided by an alter are the same as the corresponding index participant). Only index participants that distribute HIVST to alters who use a different phone number and WeChat to upload test results will receive incentives. A fixed amount of 20 RMB (≈ 3 USD) will be offered to the corresponding index participant as a financial incentive once an alters’ test result is verified. Additionally, all testers will receive a fixed 20 RMB (≈ 3 USD) as encouragement and honorarium for their time used when a CBO staff validates their results for the first time.

### Outcomes

Our first primary outcome is to compare the average number of alters motivated by each index between two groups. The second primary outcome is to ascertain the mean number of newly-tested alters motivated by each index in each group in the same study period. Secondary outcomes will be 1) the mean number of tested alters motivated by each index in each group with HIV reactive results, and 2) evaluate the primary outcomes in subgroups categorized by age, sexual orientation, sexual intercourse history, residence, and previous HIV testing experience.

### Participant timeline

We expect to start study recruitment from January 7, 2021, to January 22, 2021. Randomizing recruited participants into groups, identifying SHIs for each group, and notifying participants occurred in February 2021. The first round of HIVST orders starts from February 2021 to April 2021, and the second round of HIVST requests will be from June 2021 to July 2021. Follow-up surveys will be collected three months later following each ordering. The trial ends in October 2021.

### Sample size

We use PASS version 16.0 to calculate a study sample size of 400 index participants (200 in each group), with an alpha of 0.05 and a loss to follow-up of 20%. Our estimated sample size can provide a power of 0.80 to detect differences between groups and are calculated based on the following assumptions.

#### Assumption 1

We assumed that SHIs identified by the model could motivate one more alter on average to get tested for HIV than SHIs identified by the scale, based on the results of our previous study [21].

#### Assumption 2

Based on the results of our previous study [25], we assumed that 40% of alters motivated by SHIs identified by the scale will be new testers. We also estimate that SHIs identified by the model can motivate 0.3 more newly-tested alters than SHIs identified by scale [22].

### Recruitment

The recruitment banner ads will have a brief trial description and continue showing on Blued until a target population size of at least 2000 eligible MSM participants fills in the baseline survey. Each Blued user will only be able to finish the baseline survey once. After survey submission, eligible study participants will receive a random 8-digit verification code and the WeChat ID of SESH. All participants will be encouraged to add the SESH official WeChat and provide their unique 8-digit verified code to get 25 RMB (≈ 4 USD) as the time reimbursement of survey completion. We will add participants who do not add the SESH WeChat or send them reminder text messages according to their provided contact information in the survey. After identification, we will use Wechat or text messages to notify SHIs and ask for their verbal consent to participate in the SD-HIVST.

### Allocation and blinding

Study participants who are consented, meet inclusion criteria, and provide non-duplicate contact information (either cell-phone number or WeChat ID) will be randomly assigned to the scale group or the machine learning group by a computer-generated program with a 1:1 allocation. Only researchers will know the details of the group assignments, and study participants and CBO staff are blinded.

#### Data collection

All MSM participants will be required to complete an online survey during recruitment. Index participants will need to complete two to three surveys, including a baseline survey at their first ordering of HIVST kits, and one to two follow-up surveys based on the times of ordering. Alters will be asked to complete a survey when reporting their test results. All surveys are administered online and include electronic consent forms.

The baseline survey administered during recruitment will include questions from the following seven perspectives: sociodemographic, sexual intercourse history in the past three months, sexual health influencer scale [23], social network information, homoprejudiced violence scale [27], depression scale (patient health questionnaire-9) [28], and HIV and syphilis testing history.

The baseline survey of index participants includes additional questions from the following four perspectives: social network information, relationship with alters, experiences of secondary distribution, and social support scale [29, 30]. Questions of the survey administered to alters will include sociodemographic, sexual intercourse history in the past three months, the experience of receiving HIVST kits, previous HIV testing experience, and social network information.

#### Data management

In this trial, all surveys are self-completed, and data are stored electronically. The recruitment survey data are stored in WENJUANXING, a popular online survey tool in China. Data of index participants and alters are stored in JINSHUJU, a secure online platform where individuals can order HIVST kits, upload testing results, and fill in surveys. Only trained CBO staff and researchers will have access to JINSHUJU. We also assigned MSM participants pseudonyms following a pre-determined pattern when adding their WeChat.

#### Missing data plan

We will follow the intention-to-treat principle to conduct data analysis. Response to variables for primary and secondary outcomes analyses are required compulsory answers to complete the surveys. Therefore, we will not have any missing data in terms of analyzing the primary and secondary outcomes.

#### Data analysis

We will use descriptive analysis to report baseline characteristics and use Shapiro-Wilk’s method for data normality test. If the data has a normal distribution, a two-sample t-test will be used to calculate a 95% confidence interval (CI) to compare the differences in outcomes between the two arms. We will use a bootstrapping method in calculating the 95% CI if the data distribution is not normal. Subgroup analysis will be used to evaluate the difference between the two groups in the different subsets of index participants. We will use R version 3.6.2 software for all the data analysis. The trial is registered with the Chinese Clinical Trial Registry (ChiCTR2000039632).

## Discussion

This quasi-experimental study will evaluate the effectiveness of an ensemble machine learning model on identifying SHIs during the digital network-based secondary distribution of HIVST among MSM by comparing it with an empirical scale. Digital network-based secondary distribution leverages both social network and digital technology, and it has been proven to be feasible and can contribute to HIV testing uptake among MSM in China [25, 26]. Additionally, social network interventions for HIV prevention offer potential in the development sector, especially in this globalizing society where people are connected easily via social media and networking technology [31]. Maximizing the social network interventions by identifying the SHI might further enhance the sexual health behavior among MSM, more specifically, the distribution and uptake of HIVST. However, few studies have yet focused on it. By comparing the identification methods, we can evaluate the impact of the identified SHI, which could expand HIV testing coverage, particularly to reach the marginalized individuals or those who are reluctant to HIV and STI testing among key populations.

Nevertheless, there are a few limitations regarding ensemble machine-learning modeling and interventions that should be acknowledged. One of the limitations might be overfitting the model due to the relatively small training and testing data sets and the comparatively large prediction data set [22]. From the perspective of interventions, even if the HIVST kits are free, the setting of the deposit for each test kit may potentially impede participants from ordering and distribution, where they might have to bear the expenditure for their peers [26]. However, we believe this strategy can effectively boost the linkage to care services. In addition, due to the trial design, this HIVST ordering system is only provided to the identified SHI and limits the accessibility of all others. However, future implementation studies can resolve this by removing the restriction.

The findings of our study will have significant scientific and policy implications for the promotion of decentralized sexual health services. The study outcomes could contribute to optimizing the integrated digital network methods on HIV and STI testing. Furthermore, practical experience obtained from this implementation in real-life settings might be transferable to future initiatives to create similar self-sustaining HIV and STI testing programs among marginalized populations. If successful, similar peer-based or social network-based intervention programs can use the ensemble machine learning model for identifying the SHI to optimize outcomes.

## Trial status

We designed the study timeline to start from January 2021 to December 2021. At the time of writing this draft protocol, the recruitment process and data collection were ongoing. We will close the recruitment system on August 31, 2021, and we expect to complete all follow-ups by December 2021. Statistical analysis has not begun.

## Data Availability

Data sharing does not apply to this article as no datasets were generated or analyzed during the current study.

## Abbreviations

HIV: Human immunodeficiency virus
MSM: Men who have sex with men
SD-HIVST: Secondary distribution of HIV self-testing
SHI: Sexual health influencer
WHO: World Health Organization
SESH: Social Entrepreneurship to Spur Health
CDC: Center for Diseases Control and Prevention
Xutong: Zhuhai Xutong Voluntary Services Center
CBO: Community-based organization
STI: Sexually transmitted infection
CI: Confidence interval
